# 1596. Efficacy and Safety of Long-Acting Subcutaneous Lenacapavir in Heavily Treatment-Experienced People with Multi-Drug Resistant HIV: Week 104 Results

**DOI:** 10.1093/ofid/ofad500.1431

**Published:** 2023-11-27

**Authors:** Onyema Ogbuagu, Edwin DeJesus, Mezgebe Berhe, Gary J Richmond, Peter J Ruane, Gary I Sinclair, Moti N Ramgopal, William Sanchez, Gordon E Crofoot, Nicolas A Margot, Hui Wang, Hadas Dvory-Sobol, Martin Rhee, Jared Baeten, Sorana Segal-Maurer

**Affiliations:** Yale School of Medicine, Cheshire, Connecticut; Orlando Immunology Center, University of Central Florida College of Medicine, Orlando, FL; North Texas Infectious Diseases Consultants, Dallas, Texas; Broward Health Medical Center, Fort Lauderdale, Florida; Ruane Clinical Research, Los Angeles, California; PrismHealth NTX, Dallas, Texas; Midway Immunology & Research Center, Fort Pierce, Florida; Floridian Clinical Research, Miami Lakes, Florida; The Crofoot Research Center, Houston, Texas; Gilead Sciences Inc, Foster City, California; Gilead Sciences Inc., Foster City, California; Gilead Sciences, Foster City, California; Gilead Sciences, Foster City, California; Gilead Sciences, Foster City, California; Division of Infectious Diseases, New York–Presbyterian Queens, Flushing, New York

## Abstract

**Background:**

Lenacapavir (LEN) is a highly potent, long-acting, first-in-class inhibitor of HIV-1 capsid protein for the treatment of HIV-1 infection in adults with multidrug resistance (MDR) in combination with other antiretrovirals.

**Methods:**

CAPELLA is an ongoing, phase 2/3 study in heavily treatment-experienced people with HIV-1 with multidrug-resistance. Participants received oral LEN for loading followed by subcutaneous LEN Q6M in addition to an investigator-selected optimized background regimen (OBR). After reporting the efficacy and safety data through 52 weeks (W), the protocol was amended to allow longer follow up; we report W104 results.

**Results:**

Of 72 participants enrolled (36 in each cohort), 25% were female, 38% were Black, median age was 52 years, 64% had CD4 <200 cells/µL, and 46% had HIV-1 resistant to all 4 major classes (NRTI, NNRTI, PI, INSTI). One participant (of 72) decided not to continue in the post-W52 extension. At W104, 62% (44/71) had HIV-1 RNA <50 c/mL via FDA Snapshot algorithm, and 14% (10/71) had HIV-1 RNA >50 c/mL; 24% discontinued for reasons other than lack of efficacy (13/71) or had missing data but were on study drug (4/71). When analyzed as missing=excluded, 81% (44/54) had HIV-1 RNA <50 c/mL. CD4 count increased by a median 97 cells/µL (Q1 to Q3: 18 to 224) and the proportion of participants with CD4 count ≥200 cells/µL increased from 36% (26/72) at baseline to 71% (39/55) at W104. Fourteen participants had emergent LEN resistance (9 previously reported), 6 of whom subsequently suppressed while continuing LEN. The median (Q1–Q3) duration of follow-up on LEN was 125 (111–140) weeks. One participant discontinued due to injection site nodule at W52 (Grade 1, previously reported); no participant discontinued due to an AE after W52. Most common AEs (excluding injection site reactions [ISRs] and COVID-19) were diarrhea and nausea (19% each, +5% each since W52, respectively). LEN-related ISRs were mostly mild-to-moderate.

**Conclusion:**

In people with MDR HIV-1 and limited treatment options, LEN in combination with an OBR was well tolerated and maintained virologic suppression at W104. These data support the use of LEN for treatment of people with MDR HIV-1.

**Disclosures:**

**Onyema Ogbuagu, MD**, Gilead Sciences, Inc: Advisor/Consultant|Gilead Sciences, Inc: Honoraria|Janssen: Advisor/Consultant|Viiv: Advisor/Consultant **Edwin DeJesus, MD**, Gilead Sciences, Inc: Advisor/Consultant|Theratechnology: Advisor/Consultant|Theratechnology: Honoraria **Mezgebe Berhe, MD, MPH**, Gilead Sciences, Inc: Clinical Trial Investigator **Gary J Richmond, MD**, Gilead Sciences, Inc: Grant/Research Support|Glaxo Smith Kline: Grant/Research Support|Insmed: Grant/Research Support|TaiMed: Grant/Research Support **Peter J Ruane, MD**, Gilead Sciences, Inc: Advisor/Consultant|Viiv: Advisor/Consultant|Viiv: Honoraria **Gary I. Sinclair, MD**, Abbvie: Grant/Research Support|Gilead: Advisor/Consultant|Gilead: Grant/Research Support|Janssen: Advisor/Consultant|Janssen: Grant/Research Support|Janssen: Honoraria|Merck: Advisor/Consultant|Merck: Grant/Research Support|Merck: Honoraria|Thera: Advisor/Consultant|Thera: Grant/Research Support|Thera: Honoraria|ViiV: Advisor/Consultant|ViiV: Grant/Research Support|ViiV: Honoraria **Moti N Ramgopal, MD**, Abbvie: Honoraria|Gilead Sciences, Inc: Advisor/Consultant|Gilead Sciences, Inc: Honoraria|Janssen: Advisor/Consultant|Janssen: Honoraria|Merck: Advisor/Consultant|Viiv: Advisor/Consultant|Viiv: Honoraria **William Sanchez, MD**, Gilead Sciences, Inc: Grant/Research Support|GSK: Grant/Research Support|Merck: Advisor/Consultant|Merck: Grant/Research Support **Gordon E Crofoot, MD**, Gilead Sciences, Inc: Clinical Trial Investigator **Nicolas A Margot, MA**, Gilead Sciences, Inc: Employee|Gilead Sciences, Inc: Stocks/Bonds **Hui Wang, PhD**, Gilead Sciences, Inc: Employee|Gilead Sciences, Inc: Stocks/Bonds **Hadas Dvory-Sobol, PhD**, Gilead Sciences, Inc: Employee|Gilead Sciences, Inc: Stocks/Bonds **Martin Rhee, MD**, Gilead Sciences, Inc: Employee|Gilead Sciences, Inc: Stocks/Bonds **Jared Baeten, MD, PhD**, Gilead Sciences, Inc: Employee|Gilead Sciences, Inc: Stocks/Bonds **Sorana Segal-Maurer, MD**, Gilead Sciences, Inc: Advisor/Consultant|Gilead Sciences, Inc: Honoraria|Janssen: Advisor/Consultant|Janssen: Honoraria|Theratechnologies: Advisor/Consultant|Theratechnologies: Honoraria|Viiv: Advisor/Consultant|Viiv: Honoraria

